# The use of a 30-degree radiolucent triangle during surgery in distal avulsion fractures of the patella

**DOI:** 10.1186/s13018-023-03631-w

**Published:** 2023-03-15

**Authors:** Léa Ragot, Filippo Gerber, Xavier Lannes, Kevin Moerenhout

**Affiliations:** Department of Orthopaedics and Traumatology Surgery, Centre Hospitalier Universitaire Vaudois (CHUV), Lausanne, Switzerland

**Keywords:** Patella baja, Patella alta, Fluoroscopy, Contralateral knee, Krackow technique

## Abstract

**Background:**

Avoiding patella baja or alta after the Krackow suture technique for distal avulsion fractures of the patella can be challenging. We aim to introduce a simple and reproducible technique using a 30-degree radiolucent triangle involving the contralateral knee to ensure the correct positioning of the patella intraoperatively.

**Method:**

The radiolucent triangle is positioned under the contralateral knee before operating the injured knee. A strict lateral view is obtained using fluoroscopy as a reference before a Krackow technique is performed on the avulsion fracture of the patella.

**Results:**

The triangle technique is straightforward and easily reproducible by surgeons of all levels. It allows the surgeon to correctly position the patella intraoperatively in avulsion fracture repair and modify tension on the patellar tendon.

**Conclusion:**

This method avoids millimetric mispositioning of the operated patella, thus improving the management intraoperatively and could decrease postoperative complications.

## Background

**Fig. 1:**
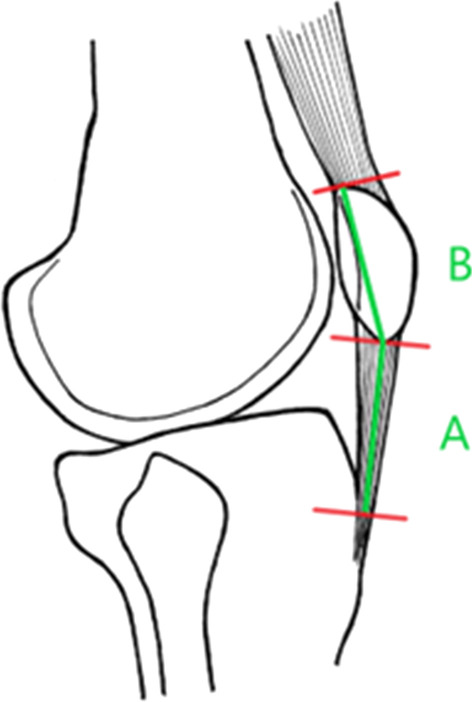
Insall-Salvati ratio = A/B

Extraarticular distal avulsion fractures of the patella generally occur in young patients (20–50-year-olds), are more frequent in males, and occur by direct trauma or with a rapid hyperflexion mechanism. They represent 5–22.4% of all patellar fractures [1] whereas patellar fractures represent approximatively 1% of all skeletal fractures [2]. Distal avulsion fractures of the patella are biomechanically equivalent to patellar tendon ruptures, which are rare, affecting < 1 per 100,000 people annually. Repairing the patellar tendon with correct positioning is crucial to avoid a severe limitation of knee flexion, early femoropatellar arthritis, and non-union [3, 4]. Patella baja or alta is diagnosed using standard orthogonal radiography before and after surgery by calculating the Insall-Salvati Ratio. A mean ratio of 1 is considered normal, a ratio of > 1.2 is diagnostic of patella alta and < 0.8 is diagnostic of patella baja [5] (Fig. 1).

However, judging the exact length of the patellar tendon intraoperatively with a Krackow technique assuring the correct positioning of the patella can be challenging. Patient positioning is an essential factor, as varying degrees of knee flexion can affect the accuracy of patellar height ratio measurement [6].

We describe a simple and reproducible technique using conventional perioperative fluoroscopy involving the contralateral knee, which to our knowledge, has not yet been described in the literature. Knee fluoroscopy is conducted on the healthy knee using a 30-degree radiolucent non sterile triangle placed under the knee to evaluate the correct position of the patella. The same radiolucent triangle, this time sterile, is used intraoperatively, allowing the operator to ensure the correct positioning and length before suturing the patella tendon.

## Method

**Fig. 2:**
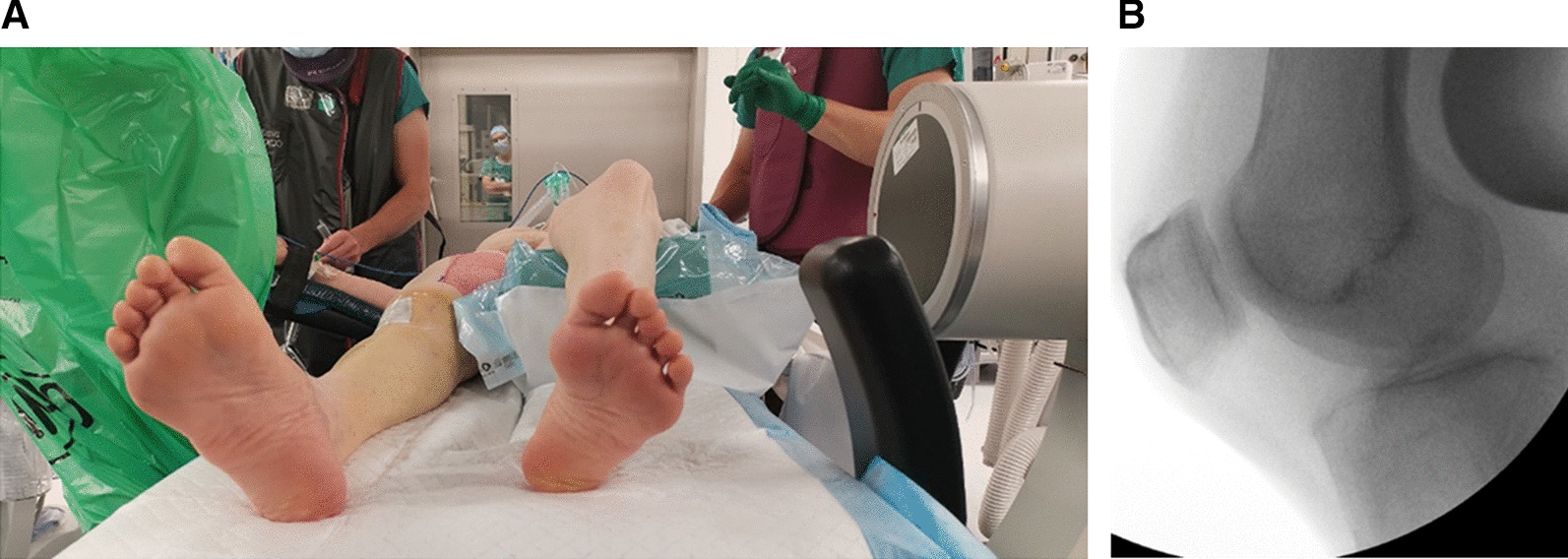
**a** patient positioning during profile fluoroscopy of the contralateral knee. **b** Lateral view of the healthy knee to reference the patella’s correct position with the 30° radiolucent triangle

**Fig. 3:**
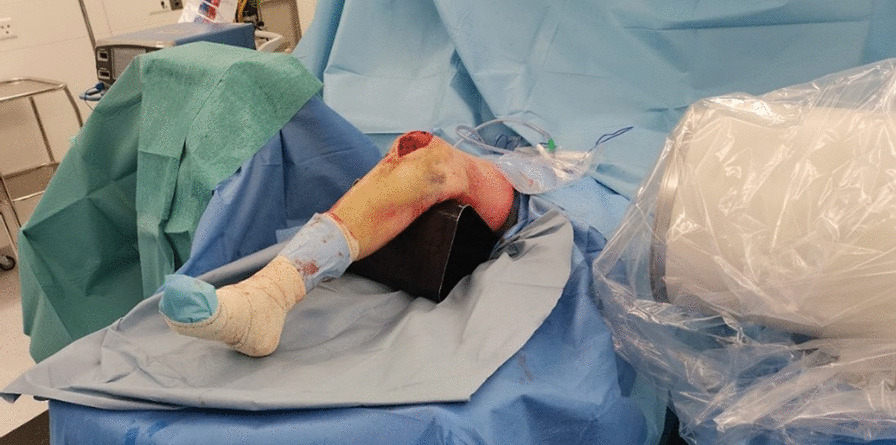
Patient positioning during final lateral view fluoroscopy of the operative knee

The radiolucent triangle at our institution, designed by INNOMED, 30/75°, 36 cm, is a convenient tool that helps position the knee with 30 degrees of flexion. The patient is supine without a bolster under the ipsilateral buttock. A general or locoregional anesthetic is administered. The radiolucent triangle is positioned under the contralateral knee, and a strict lateral view is obtained with fluoroscopy (Fig. 2a, b). Images of the lateral contralateral knee view are saved. The patient is then draped and prepped for surgery (Fig. 3).

**Fig. 4:**
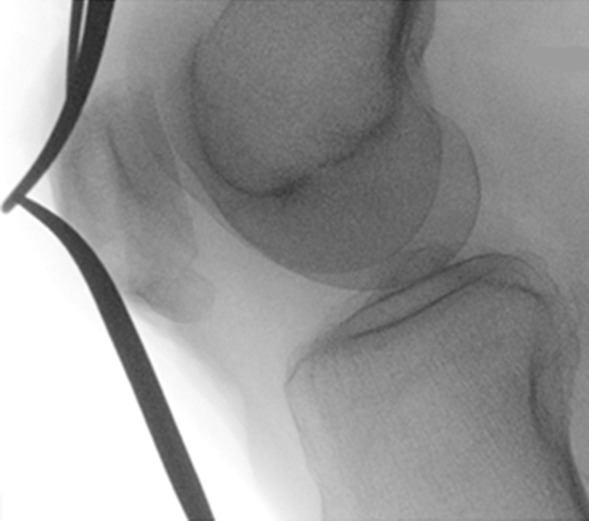
Satisfactory patellar height marked by Kelly clamps on fluoroscopy at the end of the surgical procedure

Once satisfactory images have been captured, we begin the open repair of the distal avulsion of the patella on the operative knee. The Krackow technique is frequently used at our institution and involves a continuous locking loop suture. The classic Krackow stitch involves three or more locking loops placed along each side of the ligament or tendon. Before securing the sutures, a sterile 30-degree radiolucent triangle is placed under the operative knee, thus allowing tension and positioning of the patella to be estimated on fluoroscopy and compared to the contralateral image. Suture and patellar height modifications can then be made intraoperatively (Figs. 3 and 4). Test repair stability after tying sutures is made at 90 degrees of flexion. When satisfactory patellar height and adequate contact between tendon and patella are obtained, the sutures that were previously held by clamps until satisfying height achievement can be tied, and the wound is rinsed and closed.

## Case presentation

A 78-year-old woman, known for a peripheral arterial obstructive disease treated by Aspirine Cardio 100 mg/day, consulted the Emergency department at our institution after direct trauma to her flexed right knee. The radiographs performed showed a multifragmentary distal avulsion fracture of the patella. Surgical treatment was decided, aspirin was not stopped before the surgery, and the patient underwent the operative technique described above. In our case, we used three patellar tunnels for passing our braided nonabsorbable threads (Ethibond® 6) and we put our suture knot on the superior pole of patella. We added one Ethibond® 6 in a circumferential manner to reinforce the stability.

**Fig. 5:**
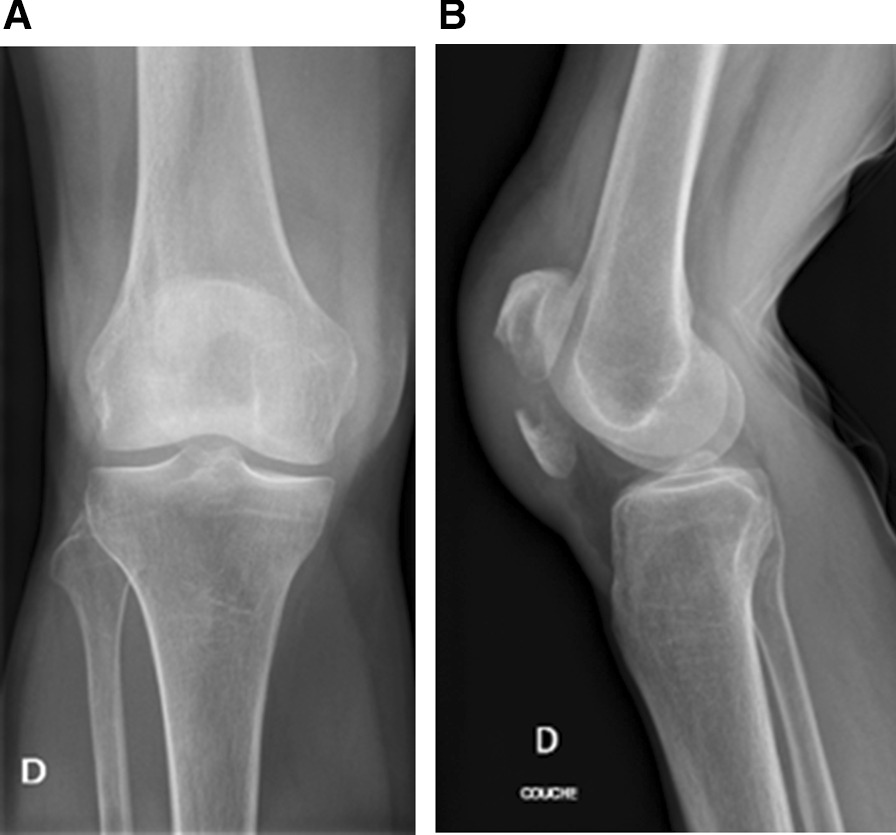
**a**, **b** Distal avulsion fracture of the right patella, x-rays before surgery

The preoperative radiographs can be seen in Fig. 5.

The patient could mobilize the operated limb from day one with a hinged brace to gradually recover the knee's range of motion using the following protocol: 0–30° for two weeks, then 0–60° from weeks three to four, then 0–90° for a final two weeks, with closed chain strengthening implying static isometric quadriceps exercises during this period, followed by free movement. Total weight bearing was authorized as of post operative day 1.

Patients can usually restart competitive sport at 4 months.

**Fig. 6:**
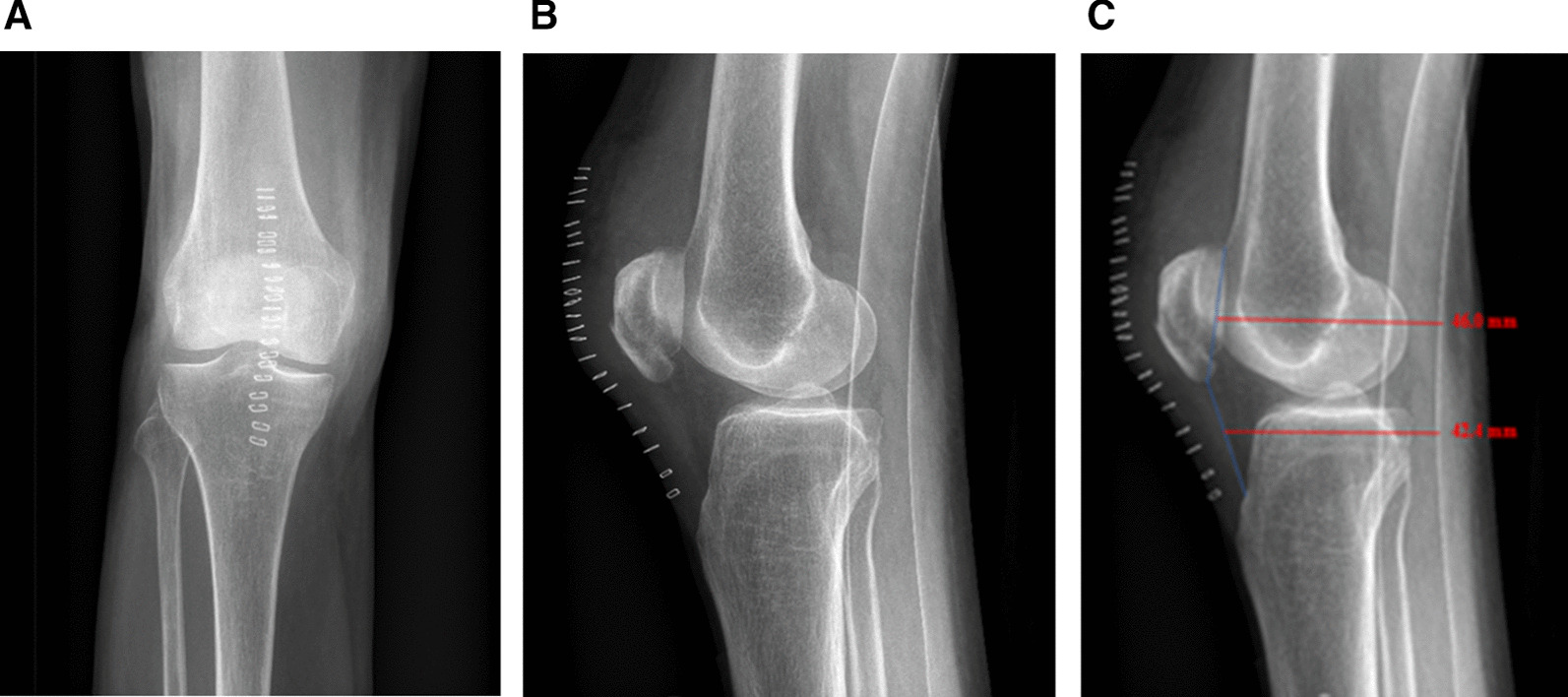
**a**, **b**, **c**: Day one post operative control x-ray—Insall-Salvati ratio = 0,92

The radiography control at day one is shown in Fig. 6.

**Fig. 7:**
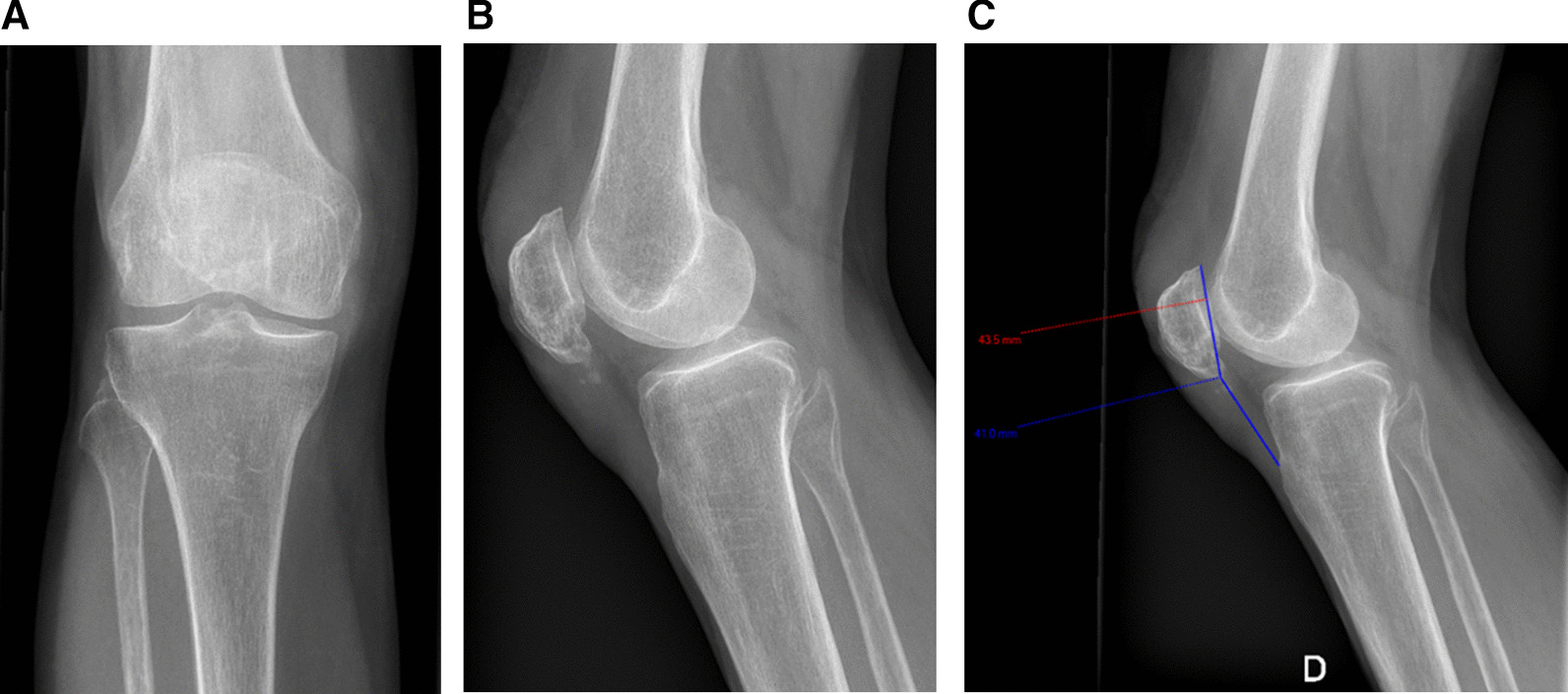
**a**, **b**, **c**: Month 6 post operative control x-ray—Insall Salvati ratio = 0,94

Both clinical and radiography results at six weeks and six months postoperative controls were satisfactory (Fig. 7).

## Discussion

The present technical note has introduced a simple technique to complement the fixation of avulsion fractures of the patella. The intraoperative 30-degree radiolucent triangle aids the surgical approach and permits correct patellar positioning. Ultimately the technique described above allows the operator to avoid patellar asymmetry via malpositioning. Further benefits include intraoperative modifications and avoiding tension problems as intraoperative feeling, as height estimations based on clinical feeling may be misleading.

Currently, priced at 390 Swiss Francs (roughly 405 Euros and 412 US dollars) and considering its aids in avoiding potentially costly complications, this technique using a resterilizable triangle is potentially very cost-effective.

Additionally, it avoids waiting for multiple conventional radiographs of the contralateral knee. These are not always available in an emergency setting and comparatively are more expensive and irradiating than one lateral fluoroscopy intraoperatively.

Our institution encourages this technique in the operative treatment of extraarticular avulsion fractures of the patella. One significant limitation is that this technique is not applicable when the contralateral knee is injured or has a pathological Insall-Salvati ratio due to previous surgery or trauma (Table 1). To avoid a misuse of our proposed technique, we present our pearls and pitfalls below (Table 2).

**Table 1:** Pros and cons of our technique using the 30-degree radiolucent triangle during surgery in distal avulsion fractures of the patella

PROS	CONS
Easy to use Reproducible Avoid patellar asymmetry via malpositioning Cost effective Less irradiating than multiple conventional radiographs	Not applicable if contralateral knee: Is injured too Has pathological Insall-Salvati ratio due to previous surgery or trauma Has previous knee arthroplasty

**Table 2:** Pearls and pitfalls of our technique

**PEARLS**
Strict profile fluoroscopy of the contralateral knee at 30° of flexion Contralateral knee positioning flush on radiotranslucent triangle Don’t forget to save the fluoroscopy image for later use Patella height analysis should be conducted before tightening sutures
**PITFALLS**
Images not conform with the requirements mentioned in the text should not be considered and may result in mal positioning of the patella

## Conclusion

Performing preoperative fluoroscopy of non-injured knees with 30 degrees of flexion using a radiolucent triangle prior to tendon repair in extraarticular avulsion fractures of the patella avoids millimetric malpositioning of the operated patella. We believe it improves intraoperative management during procedures in patella surgery and decreases postoperative complications. Further comparative studies comparing different technical procedures would be needed to confirm if this technical novelty is equivalent or better for reconstructing a comparable Insall-Salvati ratio.

## Data Availability

These are available according to the reader's request.
